# ADSC-derived exosomes mitigate radiation-induced skin injury by reducing oxidative stress, inflammation and cell death

**DOI:** 10.3389/fpubh.2025.1603431

**Published:** 2025-05-14

**Authors:** Zhe Liu, Jiawei Gu, Yakun Gao, Hao Hu, Hua Jiang

**Affiliations:** ^1^Department of Plastic Surgery, Shanghai East Hospital, School of Medicine, Tongji University, Shanghai, China; ^2^Department of Plastic Surgery, Shanghai Huashan Hospital, Fudan University School of Medicine, Shanghai, China

**Keywords:** adipose-derived stem cells, exosomes, radiation-induced skin injury, radiation-induced skin fibrosis, cell death, ROS

## Abstract

**Background:**

Radiation-induced skin injury (RISI) is a significant complication of radiotherapy and affects over 95% of patients who undergo radiation treatment. The pathophysiological cascade of RISI includes oxidative stress, persistent inflammation, and excessive fibrotic remodeling. Current treatments provide limited efficacy and primarily focusing on symptomatic relief. Exosomes from adipose-derived stem cells (ADSC-Exo) offer promising therapeutic effects on multiple types of skin injury, while their roles in the treatment of RISI remains to be fully explored.

**Method:**

A mouse model of RISI and an *in vitro* radiation-induced cellular damage model were established to evaluate the therapeutic effects of ADSC-derived exosomes. ADSC-Exo were isolated via size-exclusion chromatography and characterized using TEM, NTA, and immunoblotting. H&E staining and Masson staining were used to evaluate the extent of skin radiation-induced skin damage and fibrosis. Skin immunofluorescence was performed to assess macrophage infiltration and polarization, while immunohistochemistry staining was conducted to determine the expression levels of inflammatory mediators in the skin samples. In the *in vitro* experiments, ROS probes were used to evaluate cellular oxidative stress levels, and western blot analysis was employed to detect the expression levels of apoptosis and pyroptosis related proteins.

**Result:**

ADSC-Exo effectively alleviated radiation-induced skin injury and fibrosis, reduced macrophage infiltration, and promoted macrophage polarization toward the M2 phenotype. Additionally, ADSC-Exo decreased the expression levels of IL-1β and IL-6 in skin tissues after irradiation. In *in vitro* experiments, ADSC-Exo mitigated oxidative stress in irradiated mouse fibroblasts, and reduced the upregulation of apoptosis-related proteins BAX and CASPASE-3, as well as pyroptosis-related proteins GSDMD and CASPASE-1 after radiation exposure.

**Conclusion:**

ADSC-Exo alleviated RISI through multifaceted effects, including macrophage polarization modulation, inflammation suppression, oxidative stress reduction, and inhibition of apoptosis and pyroptosis. These findings support the potential of ADSC-Exo as a promising cell-free therapy for RISI.

## Introduction

Radiation-induced skin injury (RISI) remains a clinically significant complication of radiotherapy. In developed countries such as those in Europe and North America, more than 70% of cancer patients require radiotherapy for their treatment (1). Among these patients, over 95% experience varying degrees of radiation-induced skin damage (2). Erythema occurs in more than 90% of these patients, followed by moist desquamation in more than 30% of patients. The risk factors including intrinsic factors such as age, sex, smoking, poor nutritional status, high body mass index, etc. as well as extrinsic factors, such as coexisting disease, total radiation dose, the dose fractionation schedule, etc. Severe RISI can progress into irreversible conditions such as skin fibrosis, chronic skin ulcers, and even radiation-induced skin cancers. These complications not only cause immense physical and psychological suffering to patients but also hinder the continuation of primary disease treatments.

The pathological cascade of RISI involves complex biological processes, including persistent inflammation, oxidative stress-induced tissue damage, and progressive fibrotic remodeling (3). Current therapeutic approaches, including corticosteroids and topical emollients, are focused on symptomatic relief rather than addressing the root molecular mechanisms of injury (4). Furthermore, these interventions offer only limited efficacy and are often associated with adverse side effects. These facts highlight an urgent need for the development of mechanism-based therapies capable of targeting multiple pathological pathways involved in RISI, providing both effective treatment and improved patient outcomes.

Recent advancements in regenerative medicine have underscored the therapeutic potential of mesenchymal stem cells (MSCs) and their secretory extracellular vesicles, particularly exosomes, in promoting tissue repair and modulating inflammation (5, 6). Notably, the use of exosomes offers significant advantages over direct MSC-based therapies by addressing critical issues such as ethical concerns, tumorigenic risks, and challenges related to storage, handling, and transportation (7). Among various types of MSCs, adipose-derived stem cells (ADSCs) have gained considerable attention due to their abundant availability, minimally invasive harvesting procedures, and robust paracrine activity (8). The ADSC-derived exosomes (ADSC-Exo) has been demonstrated to have exceptional capabilities in reducing inflammation, scavenging reactive oxygen species (ROS), and enhancing angiogenesis in various disease models (9). Moreover, several studies have confirmed the efficacy of ADSC-Exo in promoting skin wound healing, including in cases of mechanical skin injuries and chronic wounds (10, 11). Nonetheless, considering the intricate mechanisms underlying RISI, the therapeutic potential of ADSC-Exo in its treatment, as well as the precise pathways through which these effects are mediated, have yet to be comprehensively elucidated.

In this study, we established a mouse model of radiation-induced skin injury and a radiation-induced cellular damage model. We demonstrated that ADSC-Exo effectively alleviated radiation-induced skin injury through multiple mechanisms, including the attenuation of radiation-induced skin fibrosis, regulation of macrophage polarization, mitigation of inflammatory responses, reduction of oxidative stress, and alleviation of cell death. These results highlight the significant therapeutic potential of ADSC-Exo in the treatment of RISI.

## Materials and methods

### Animals and radiation-induced skin injury model

Male C57BL/6 mice weighing 21–23 g were purchased from Shanghai Jihui Laboratory Animal Care Co. Animals were maintained under a 12 h light/dark cycle in a temperature-controlled facility with unrestricted access to water and standard rodent chow.

To establish a RISI mice model, mice were anesthetized with atomized isoflurane and quickly fixed in a radiation-specific acrylic container in a prone position. 5 cm thick lead bricks were used to shield the mice head and body below the neck, and a ^60^Co irradiation device was used as the radiation source to administer a single dose of 30 Gy ionizing radiation to the mice. The dose rate was 0.87 Gy/min.

All the animal procedures were in accordance with the Guide for the Care and Use of Laboratory Animals and approved by the ethics review board of Tongji University School of Medicine.

### Cell culture and cell irradiation model

Adipose derived stem cells (ADSCs) were isolated and cultured according to established protocol (12). Briefly, Testicular adipose tissues were collected from healthy C57BL/6 mice. The tissues were washed with PBS and cut into approximately 1 mm^3^ fragments. Digestion was performed using 2 mg/mL collagenase type I (OriCell, COLA-10001-50) at 37°C for 40 min with gentle agitation. The enzymatic digestion was terminated by adding an equal volume of complete culture medium (DMEM-F12 supplemented with 10% fetal bovine serum and 1% penicillin–streptomycin). Then, 70 μm cell strainer was used to remove debris, and Red Blood Cell Lysis Buffer (Beyotime, China) was used to remove red blood cells. The resultant cells were seeded in six-well plate and cultured at 37°C with 5% CO₂. Once adherent cells reached 80–90% confluence, they were passaged at a ratio of 1:2 or 1:3. ADSCs at passage 3 were used for subsequent experiments.

To confirm the purity of ADSCs, immunofluorescence staining was performed to detect the expression of ADSCs markers CD44 and CD105. In addition, flow cytometry was conducted to examine the expression of the ADSCs marker CD90 and the hematopoietic cell marker CD45.

The murine fibroblast cell line L929 was obtained from the Nation Collection of Authenticated Cell Cultures. Cells were cultured in MEM cell culture medium (Gibco, 11,095,080) supplemented with 10% horse serum (Gibco, 26,050,088) and 1% antibiotic-antimycotic solution (Beyotime, C0224).

### Isolation and characterization of ADSC-derived exosomes

ADSC-derived exosomes were isolated based on size-exclusion chromatography (SEC) method, according to the protocol. Briefly, when ADSCs reached 80–90% confluence, the FBS-free culture medium was changed and maintained for 48 h. The cell culture supernatant was filtered with 0.22 μm membrane filters and mixed with an equal volume of exosome concentration reagent (Servicebio, G4114). The mixture was vortexed vigorously for 15–60 s and incubated at 4°C for 2 h. Subsequently, the mixture was centrifuged at 10,000 × g for 60 min at 4°C. The supernatant was discarded, and the precipitate was resuspended in PBS at a ratio of 1:100 to obtain an exosome concentrate. Next, 0.5 mL of the concentrated exosome sample was loaded onto an exosome isolation chromatography column (Servicebio, G4112). Once the sample had completely entered the column, PBS was added to wash the column, and a 1 mL eluate was collected. This eluate was rich in exosomes used for subsequent experiments.

The protein concentration of exosomes was determined using the bicinchoninic acid assay (Servicebio, G2026-200 T), Western blotting assay were used to detect the exosomes markers CD63 (Servicebio, GB115712) and CD81 (Servicebio, GB111073). The morphology and size distribution of the exosomes were analyzed using transmission electron microscopy (TEM) and nanoparticle tracking analysis (NTA), respectively.

### ADSC-Exo treatment

For mice ADSC-Exo treatment, A total of 50 μg of exosomes were diluted in 150 μL of sterile PBS. Immediately after irradiation, using an insulin syringe, 150 μL of diluted exosomes were evenly injected into the dermis layer of the skin on the mouse’s neck at six points. The Control group and IR group were injected with the equal volume of PBS using the same method.

For cell ADSC-Exo treatment, immediately after irradiation, the cells were treated with ADSC-Exo at a concentration of 25 μg/mL, while the control group and the IR group were administered an equal volume of sterile PBS.

### H&E staining, Masson staining and immunohistochemistry staining

Ten days post-irradiation, the mice were euthanized using the carbon dioxide (CO₂) method, and a 0.5 cm^2^ full-thickness skin tissue sample was collected from the cervical region. The samples were fixed in 4% paraformaldehyde, gradually dehydrated using a graded ethanol series, embedded in paraffin, and sectioned into 5 μm-thick slices. Histological changes and collagen deposition were evaluated using hematoxylin and eosin (H&E) staining and Masson’s trichrome staining kits, following the manufacturer’s instructions.

For IHC staining, the sections were immersed in 3% hydrogen peroxide (H₂O₂) at 37°C for 15 min to inactivate endogenous peroxidase activity. They were then blocked with 5% bovine serum albumin (BSA) in PBS for 1 h to prevent non-specific binding. The slides were subsequently incubated overnight at 4°C with primary antibodies against IL-1β (Cell Signaling Technology, 12,242) and IL-6 (Cell Signaling Technology, 12,912). On the following day, the slides were treated with diaminobenzidine (DAB) for visualization. IHC-positive scores were quantified using ImageJ software with the IHC Toolbox plugin.

### Immunofluorescence staining

Sample preparation and preprocessing were performed as described previously. Paraffin sections (10 μm thick) were rehydrated in PBS for 10 min, followed by permeabilization in pre-chilled methanol at −20°C for 30 min. To block non-specific binding, the sections were treated with a blocking solution containing 0.3% Triton X-100, 1% BSA, 1% FBS, and 0.1 M Tris–HCl supplemented with goat serum. The sections were then incubated overnight at 4°C with primary antibodies against F4/80 (Abcam, ab6640), CD86 (Abcam, ab317266), and CD206 (Abcam, Ab64693). On the following day, sections were incubated with fluorescent secondary antibodies specific to the primary antibodies’ species, and nuclei were counterstained with 4′,6-diamidino-2-phenylindole (DAPI, Servicebio, G1012). Fluorescent images were analyzed using ImageJ software.

### Western blot assay

Cells or exosome samples were lysed in RIPA lysis buffer (Servicebio, G2002) containing protease inhibitors (Selleck, B14001). Protein separation was performed using 10% or 15% SDS-PAGE, followed by transfer onto polyvinylidene fluoride (PVDF) membranes (Merck Millipore, K5MA6539B). Membranes were blocked with 5% BSA and incubated overnight at 4°C with primary antibodies, including anti-CASPASE-3 (Cell Signaling Technology, 9,662, 1:1000), anti-CASPASE1 (Cell Signaling Technology, 24,232, 1:1000), anti-GSDMD (Cell Signaling Technology, 39,754, 1:1000), anti-BAX (Cell Signaling Technology, 2,772, 1:1000), and anti-GAPDH (Abclonal, A19056, 1:20000). After three washes with TBST, membranes were probed with HRP-conjugated secondary antibodies at room temperature for 1 h. Protein bands were visualized using an enhanced chemiluminescence detection kit (Millipore Corporation, MA01821).

### Measurement of cellular ROS

Intracellular ROS levels were measured using the DCFH-DA probe according to the manufacturer’s protocol (Beyotime, S0033S). Briefly, 4 h after L929 cell irradiation, DCFH-DA was diluted in PBS to a final concentration of 10 μM. The cell culture medium was removed, and cells were incubated with the diluted DCFH-DA probe at 37°C for 20 min. Following three washes with PBS, fluorescence images were captured using a Zeiss fluorescence microscope.

### Statistical analysis

Statistical analysis was conducted using GraphPad Prism software (v 9.0). Data are expressed as the mean ± SD from at least three biologically independent samples. For comparisons between two groups, an unpaired two-tailed Student’s *t*-test was applied, while a two-way analysis of variance (ANOVA) was used for comparisons involving three or more groups. A *p*-value < 0.05 was considered indicative of statistical significance.

## Results

### The identification of ADSCs and ADSC-Exo

**Figure 1:**
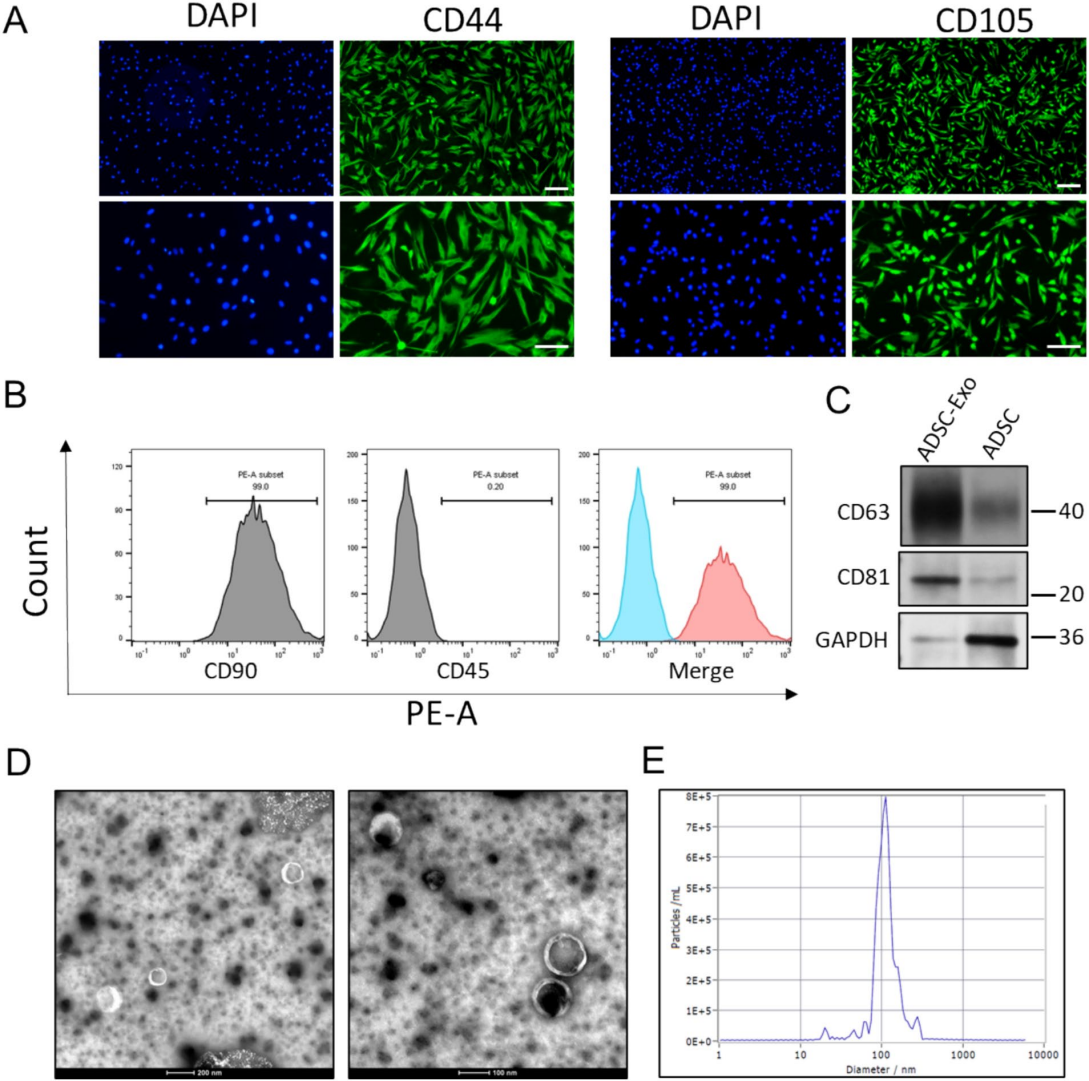
The identification of ADSC and ADSC-Exo. **(A)** Immunofluorescence images show positive staining for CD44 and CD105 in ADSC cells. Scale bar = 100 μm (up) and scale bar = 50 μm (down). **(B)** Flow cytometry analysis demonstrates that ADSC cells are positive for the mesenchymal stem cell marker CD90 and negative for the hematopoietic marker CD45. **(C)** Western blots assay showed the expression of exosome markers CD63 and CD81. **(D)** Transmission electron microscope (TEM) scanning image showed the morphology of ADSC-Exo. **(E)** Nanoparticle tracking analysis showed the size distribution of isolated ADSC-Exo.

Adipose-derived stem cells (ADSCs) exhibited a spindle-shaped morphology characteristic of mesenchymal lineages. Immunofluorescence staining revealed positive expression of ADSCs markers CD44 and CD105 (Figure 1A). Flow cytometric assay demonstrated pronounced expression of canonical ADSCs markers CD90 (>95%), with minimal detection of hematopoietic lineage markers CD45 (<0.5%) (Figure 1B), confirming adherence to established ADSC immunophenotypic criteria.

The ADSCs were then cultured in FBS-free medium for exosomes release. Size-exclusion chromatography (SEC) method was employed for exosomes isolation. Immunoblotting verified enriched expression of exosome-specific markers (CD63 and CD81), with minimal detection of the cytoplasmic marker GAPDH (Figure 1C). Transmission electron microscopy (TEM) identified nanovesicles with a cup-shaped morphology and bilayer membrane structure (Figure 1D). Nanoparticle tracking analysis (NTA) quantified a predominant size distribution peaking at 109.9 nm (Figure 1E), consistent with typical exosomes dimensions. These results collectively confirmed successful purification of ADSC-derived exosomes (ADSC-Exo).

### ADSC-Exo alleviated radiation-induced skin fibrosis

**Figure 2:**
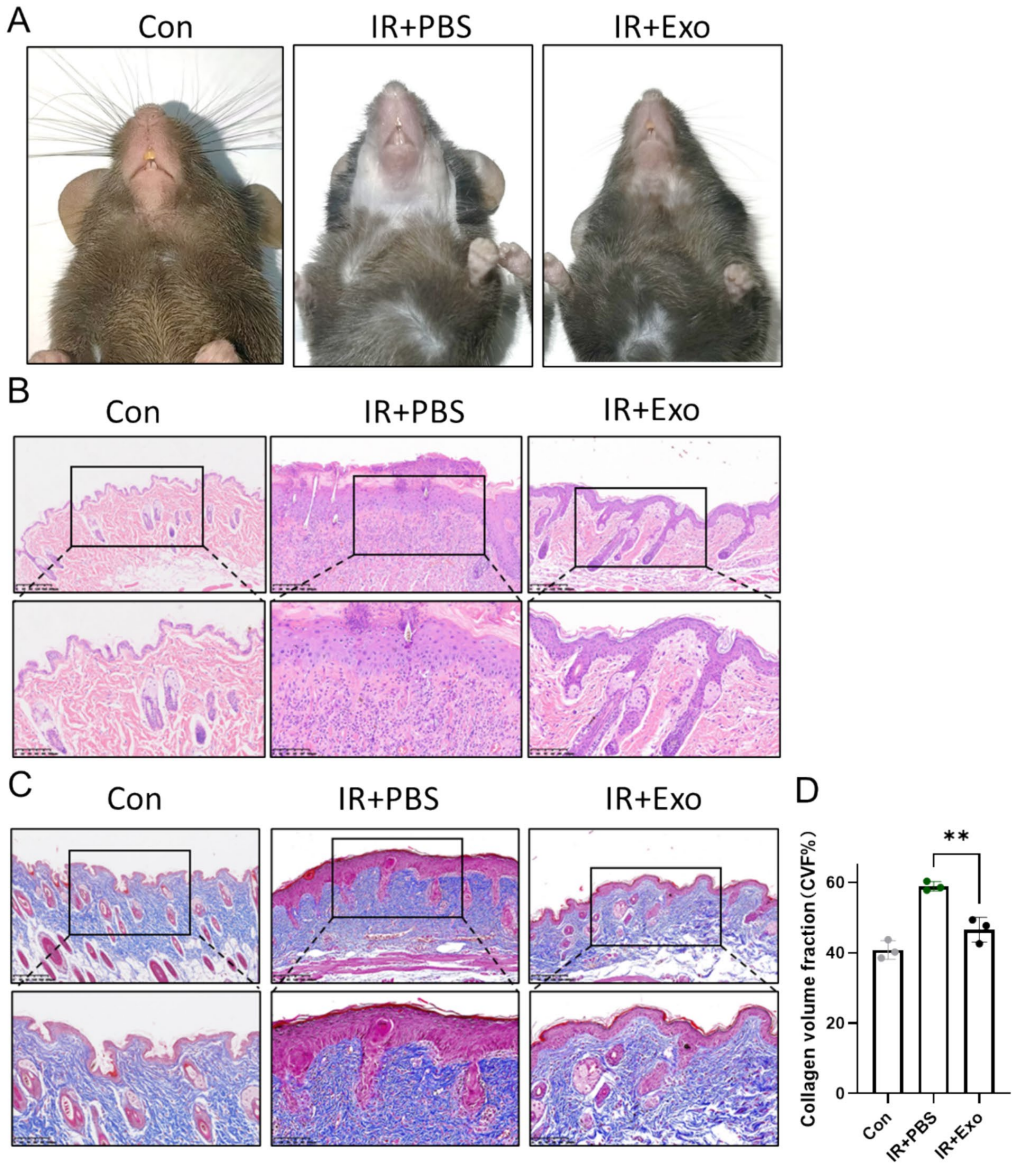
ADSC-Exo alleviated RISI and RISF. **(A)** Photos showed the morphological change of mice cervical skin in each group 10 days post irradiation. **(B,C)** H&E staining **(B)** and Masson staining **(C)** of mice cervical skin in each group 10 days post irradiation. Scale bar = 200 μm (up) and scale bar = 100 μm (down), *n* = 3. **(D)** Quantification of the collagen volume fraction (%) of mice cervical skin in each group 10 days post irradiation (*n* = 3). Error bars are presented as mean ± SD, ** means *p* < 0.01.

To further investigate the therapeutic role of ADSC-Exo in radiation-induced skin injury, a murine model of cervical radiation injury was established using a single 30 Gy ionizing radiation. In the IR + Exo group, 50 μg of exosomes per mouse were administered via multi-point subcutaneous injections in the cervical region every 2 days, while the irradiated control group (IR + PBS group) received equal volumes of PBS. At 10 days post-irradiation, the IR + PBS group exhibited typical symptoms of radiation-induced skin damage, including pronounced alopecia, desquamation, and erythema, which were markedly alleviated by ADSC-Exo treatment (IR + Exo group) (Figure 2A). Histopathological analysis revealed epidermal hyperplasia, dermal disorganization, appendage loss, and inflammatory cell infiltration in IR + PBS group, whereas the IR + Exo group exhibited mild histopathological alterations in the skin with significantly reduced inflammatory cell infiltration (Figure 2B). Masson staining further demonstrated substantial subcutaneous collagen deposition in the IR + PBS group, indicating pronounced radiation-induced skin fibrosis, which was also attenuated by ADSC-Exo treatment (Figures 2C,D). Collectively, these findings provide preliminary evidence that ADSC-Exo mitigates radiation-induced skin damage and fibrotic remodeling.

### ADSC-Exo attenuated radiation-induced macrophage infiltration and promoted macrophage M2 polarization

**Figure 3:**
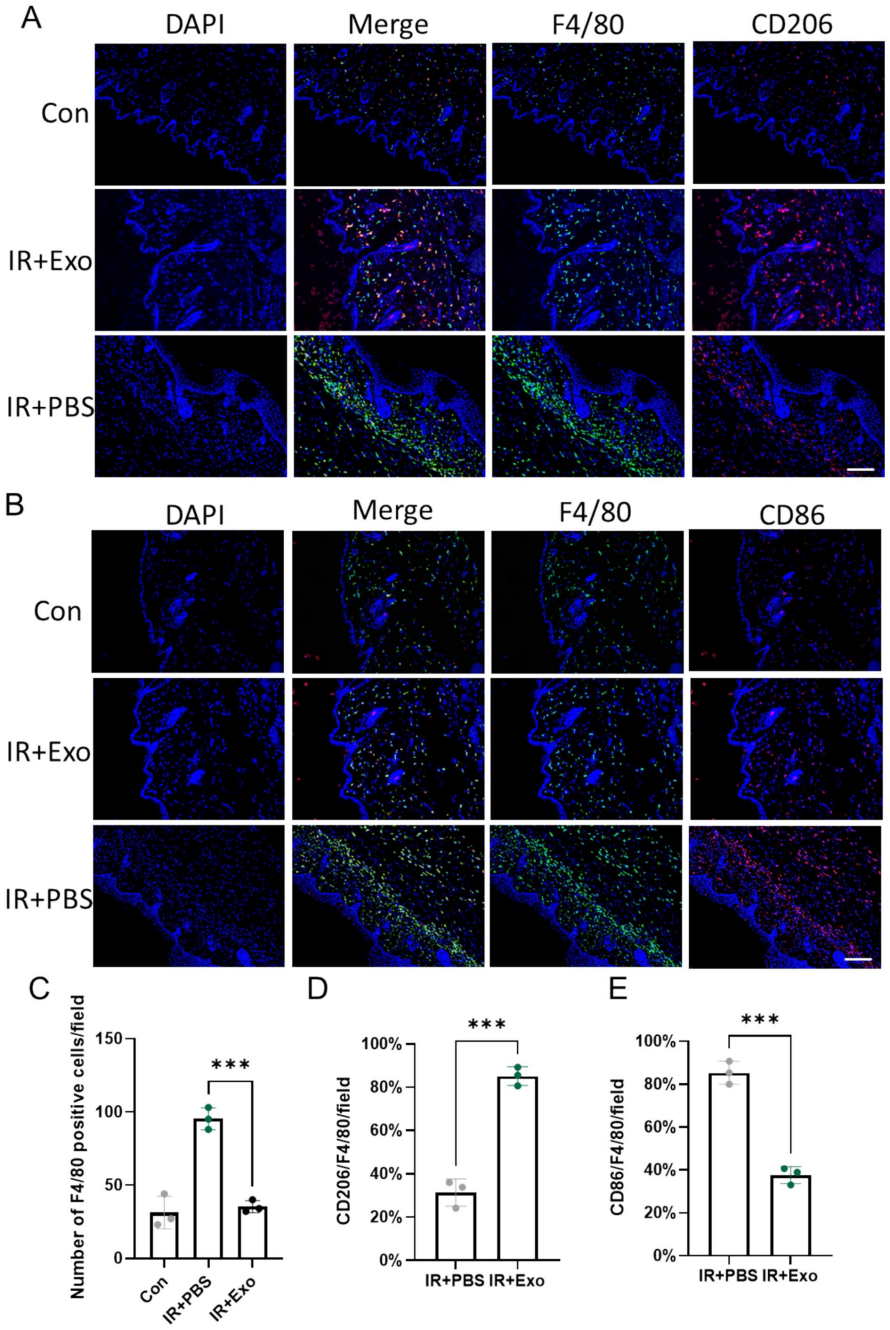
ADSC-Exo attenuated radiation-induced macrophage infiltration and promoted macrophage M2 polarization. **(A)** Immunofluorescence images show F4/80-positive cells (green) and CD206-positive cells (red) in the cervical skin of mice at 10 days post irradiation. Scale bar = 100 μm, *n* = 3. **(B)** Immunofluorescence images show F4/80-positive cells (green) and CD86-positive cells (red) in the cervical skin of mice at 10 days post irradiation. Scale bar = 100 μm, *n* = 3. **(C)** Quantification of F4/80 positive cells pre field in each group, Error bars are presented as mean ± SD, *n* = 3, *** means *p* < 0.001. **(D,E)** The bar plot showed the proportion of CD206-positive cells to F4/80-positive cells **(D)** or the proportion of CD86-positive cells to F4/80-positive cells **(E)** in different groups, *n* = 3. Error bars are presented as mean ± SD, *** means *p* < 0.001.

Macrophages are pivotal immune cell types in skin tissue. During skin injury repair, macrophages are recruited to the injury site, where they phagocytose dead cells and regulate localized inflammatory responses and collagen deposition. Macrophage polarization is one of the primary mechanisms underlying their functional diversity. We observed that, compared to the control group (Con group), substantial macrophages infiltration occurred in the dermis and subcutaneous tissue at 10 days post-radiation (IR + PBS group) (Figures 3A,C), with a high proportion of macrophages expressing the M1 macrophage marker CD86 (Figures 3B,E), indicating a predominance of pro-inflammatory M1-like macrophages during the early phase of radiation injury. ADSC-Exo treatment significantly reduced macrophage infiltration and promoted their polarization toward M2-like macrophages (Figures 3A,D), marked by high expression level of CD206. These findings suggest that ADSC-Exo plays a critical role in the reduction of macrophage infiltration and driving macrophage conversion toward an anti-inflammatory phenotype.

### ADSC-Exo attenuate radiation-induced skin inflammation

**Figure 4:**
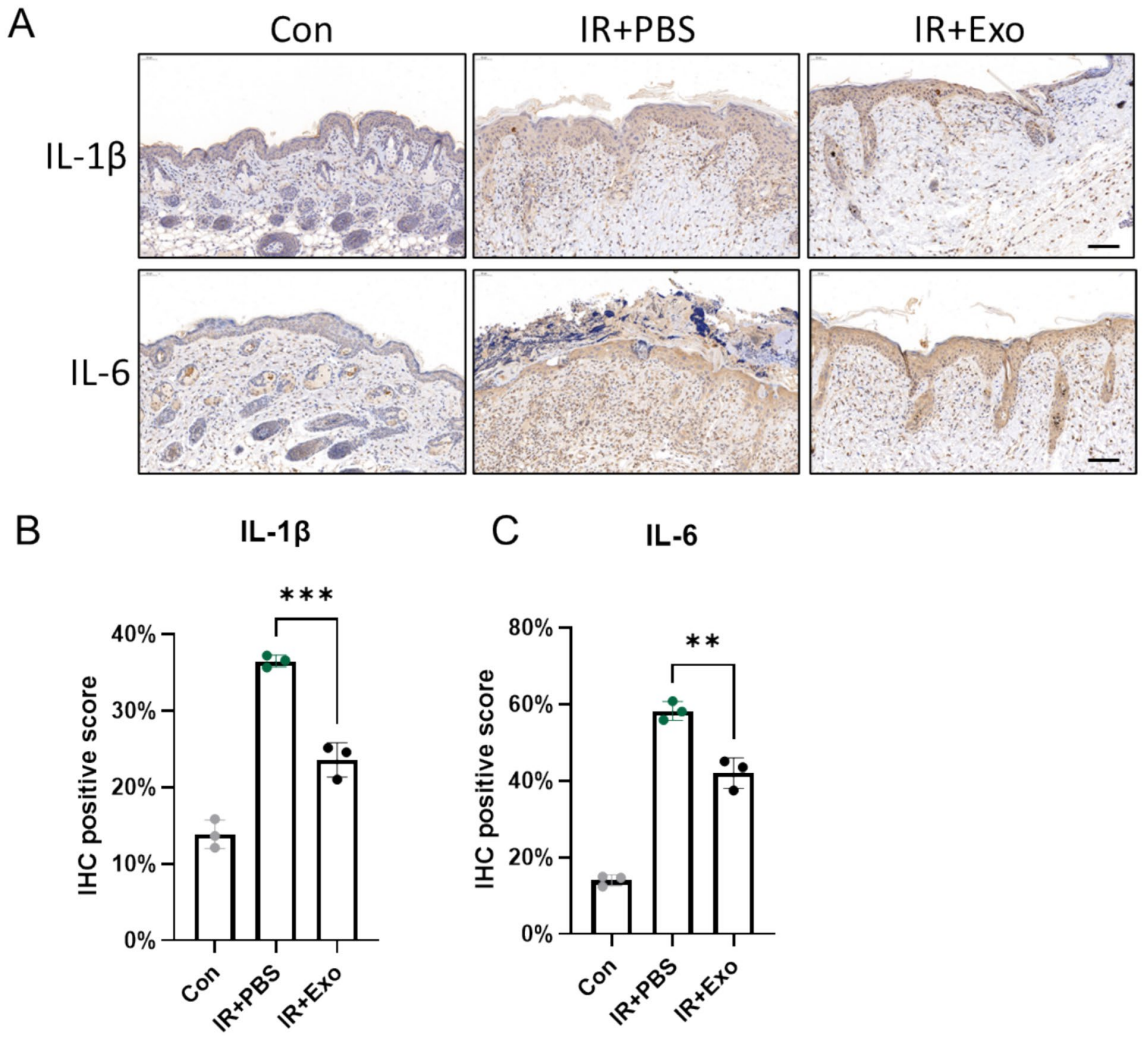
ADSC-Exo attenuate radiation-induced skin inflammation. **(A)** IHC staining revealed the expression level of IL-1β and IL-6 in mice cervical skin at 10 days post-irradiation. Scale bar = 100 μm, *n* = 3. **(B,C)** The quantification of IL-1β **(B)** and IL-6 **(C)** positive score in each group, *n* = 3. Error bars are presented as mean ± SD, ** means *p* < 0.01, *** means *p* < 0.001.

Radiation-induced inflammatory responses provoke excessive release of pro-inflammatory cytokines, which exacerbate tissue damage by disrupting epidermal integrity, promoting dermal disorganization, and amplifying oxidative stress. We observed significantly elevated expression levels of IL-1β and IL-6 in irradiated skin tissues (IR + PBS group) at 10 days post-irradiation compared to controls group (Con group), with pronounced IL-1β and IL-6 positive staining localized in both the epidermis and dermis, indicative of robust radiation-induced inflammatory responses. ADSC-Exo treatment (IR + Exo group) markedly attenuated radiation-triggered upregulation of IL-1β and IL-6, particularly within the dermal (Figure 4A). Consistently, IHC positivity score for these cytokines were significantly reduced in the IR + Exo group compared to the IR + PBS group (*p* < 0.01) (Figures 4B,C). These findings collectively suggested that ADSC-Exo alleviates radiation-induced skin injury through suppression of pro-inflammatory signaling.

### ADSC-Exo alleviated radiation-induced ROS accumulation, cell apoptosis and pyroptosis

**Figure 5:**
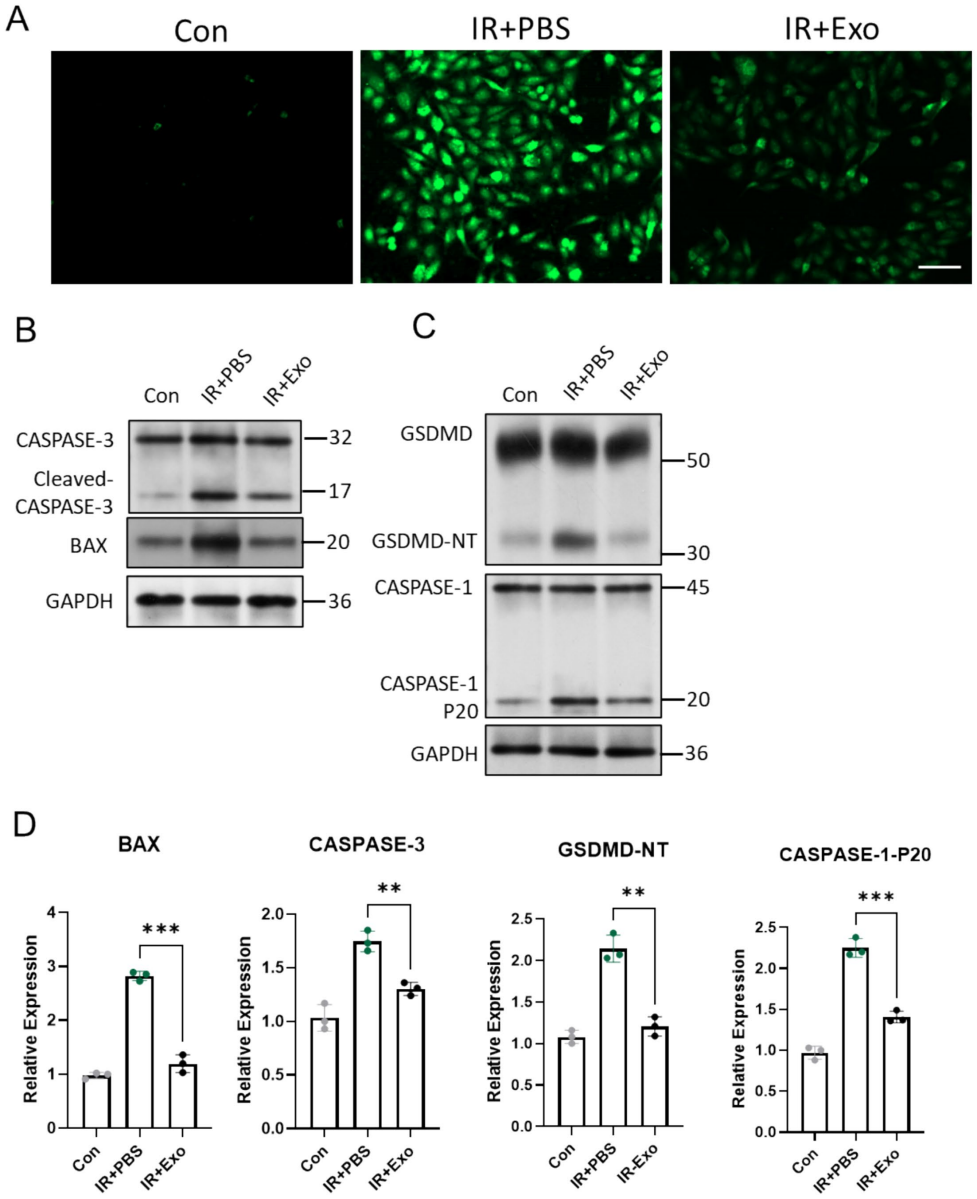
ADSC-Exo alleviated radiation-induced ROS accumulation, cell apoptosis and pyroptosis. **(A)** Fluorescence images show intracellular ROS levels in different groups 6 h after irradiation or without irradiation. Scale bar = 100 μm. **(B,C)** Western blot assay revealed the expression level of CASPASE-3, BAX, GSDMD and CASPASE-1. **(D)** The quantification of gray value of indicated proteins/ GAPDH, *n* = 3. Error bars are presented as mean ± SD, ** means *p* < 0.01, *** means *p* < 0.001.

Radiation induced tissue damage Primarily through indirect mechanisms, notably by triggering excessive generation of reactive oxygen species (ROS), which drives multiple forms of cell death (13). Fibroblasts serve as critical cellular components of the dermal layer, and play essential roles in maintaining cutaneous homeostasis, orchestrating injury repair, and modulating inflammatory responses. We observed that L929 murine fibroblasts exposed to 12 Gy irradiation (IR + PBS group) exhibited markedly elevated intracellular ROS levels compared to controls (Con group) at 6 h post radiation exposure, whereas ADSC-Exo treatment (IR + Exo) significantly attenuated radiation-induced ROS accumulation, demonstrating its potent antioxidant capacity (Figure 5A). Immunoblotting analysis further revealed substantial upregulation of key mitochondrial apoptotic-related protein (BAX and cleaved Caspase-3) and pyroptotic-related protein (GSDMD and Caspase-1-P20) in irradiated L929 cells at 48 h post-irradiation. ADSC-Exo treatment suppressed the radiation-triggered upregulation of these and pyroptotic mediators (Figures 5B–D). Collectively, these findings suggested that ADSC-Exo mitigates radiation-induced fibroblast apoptosis and pyroptosis, potentially *via* its ROS scavenging-dependent mechanisms.

## Discussion

Radiation-induced skin injury represents a significant clinical challenge in radiotherapy, particularly affecting patients undergoing radiotherapy for head, neck, and breast cancers. This iatrogenic complication manifested as acute dermatitis and often progressed into chronic fibrosis or skin ulcer. The pathological cascade involves persistent inflammation, oxidative stress, DNA damage, cell death, and aberrant tissue remodeling. Currently, there is no universally recognized gold standard treatment for RISI in clinical practice. To date, several commercially available topical ointments have been developed, primarily containing small-molecule drugs designed to chemically neutralize toxic free radicals within skin cells. However, their therapeutic efficacy remains constrained by inherent shortcomings, including poor solubility, limited chemical stability, and the potential side effects, etc. (14). Therefore, addressing these challenges is imperative to improve the therapeutic outcomes for RISI.

Our study demonstrates that ADSC-Exo represents an effective therapeutic approach for RISI. Exosomes are actively secreted extracellular vesicles with diameters ranging from 30 to 200 nm. They possess a lipid bilayer structure and are rich in biological macromolecules such as mRNA and proteins. Exosomes can easily cross biological barriers, including the blood–brain barrier and cellular membranes. These characteristics make exosomes a promising therapeutic delivery vehicle (15, 16). Compared to directly stem cell treatment, exosomes derived from stem cells offer significant advantages in clinical administration. Being cell-free, exosomes mitigate the risks of tumorigenicity and immune rejection associated with stem cell injection. Additionally, exosomes exhibit superior stability, easier storage, and the ability to be precisely engineered for delivering specific therapeutic molecules, making them a safer and more versatile alternative for clinical application (17). A recent study indicated that adipose-derived stem cells (ADSCs) exert their therapeutic effects on RISI primarily through paracrine mechanisms, further underscoring the critical role of exosomes in the therapeutic process (18). Compared with other types of mesenchymal stem cell, ADSCs offer distinct advantages due to their abundance, ease of accessibility, and minimal invasiveness during harvest, typically via liposuction (19). Moreover, ADSCs exhibit high proliferative capacity and secrete exosomes enriched with bioactive molecules such as growth factors, cytokines, and antioxidative components, which are highly effective in tissue repair and inflammation resolution (20).

Several studies have reported that mesenchymal stem cell-derived exosomes (MSC-Exo) facilitate the repair of skin injury through diverse mechanisms, including the inhibition of fibroblast activation, promotion of re-epithelialization, enhancement of angiogenesis, and attenuation of inflammation (21–23). These findings highlight the multifunctionality of MSC-derived exosomes. Comparing with mechanical skin injuries, RISI is associated with more severe cell death, oxidative stress, and inflammatory responses. Current researches suggest that excessive production of reactive oxygen species (ROS) is a primary mechanism underlying radiation-induced tissue damage. Excessive ROS leads to the denaturation of biological macromolecules, causing DNA damage and subsequent cell death (24). Studies have shown that MSC-Exo may alleviate radiation-induced oxidative stress damage by activating the NRF2 pathway (25). Additionally, our study confirmed that ADSC-Exo alleviates radiation-induced apoptosis and pyroptosis in dermal fibroblasts. Among the various forms of cell death, pyroptosis tends to trigger a stronger inflammatory response, which further promotes excessive recruitment of immune cells and local ROS production, creating a vicious cycle (26). Critical mediators of pyroptosis, such as IL-1β and IL-6, can amplify inflammatory cascades and promote fibrosis (27, 28). The ability of ADSC-Exo to inhibit pyroptosis has also been confirmed by other recent investigations, exosome/circHIPK3/ FOXO3a pathway was reported as a critical pathway in alleviating pyroptosis (29, 30). Thus, we propose that the ROS-reducing effects and pyroptosis-ameliorating effects of ADSC-Exo may play a more critical role in the treatment of RISI.

Furthermore, macrophage polarization is the basis for exerting its distinct biological functions, with M1 macrophages promoting inflammation and M2 macrophages driving tissue repair and regeneration. Several studies have suggested that MSC-derived exosomes modulate macrophage polarization through mechanisms such as the transfer of miRNAs or functional proteins (31). Studies have shown that MSC-Exo can target the P38 MAPK/NF-κB pathway to facilitate M2 polarization and reduce the M1-to-M2 polarization ratio (32). In our study, radiation-induced skin damage resulted in significant infiltration of pro-inflammatory M1 macrophages. The imbalanced polarization of macrophages toward the M1 phenotype further exacerbates local ROS level and inflammatory responses, leading to aggravated tissue damage (33). Therefore, the regulatory effect of exosomes on macrophage polarization may serve as an essential basis for their therapeutic role in RISI.

Despite the promising result, several issues remain to be addressed. First, this study primarily focused on animal models, and the efficacy of ADSC-Exo in treating RISI needs further validation in clinical experiments. Second, the specific molecular mechanisms through which ADSC-Exo exert their therapeutic effects remain to be fully elucidated. Additionally, the development of more efficient exosomes isolation and production methods is necessary to meet the growing demand for large-scale clinical applications.

In conclusion, our study demonstrates the multifaceted therapeutic potential of ADSC-Exo in mitigating RISI. Specifically, ADSC-Exo alleviated oxidative stress, reduced apoptosis and pyroptosis, attenuated fibrosis and inflammation, and modulated macrophage polarization. These findings highlighted ADSC-Exo as a promising and innovative therapeutic strategy for the management of RISI.

## Data Availability

The raw data supporting the conclusions of this article will be made available by the authors, without undue reservation.
